# A Network Investigation on Idiopathic Hypogonadotropic Hypogonadism in China

**DOI:** 10.1155/2013/591012

**Published:** 2013-12-09

**Authors:** Weiwei Zhao, Hongying Ye, Xiaolong Zhao, Zhaoyun Zhang, Shouyue Sun, Yiran Jiang, Min He, Cheng Xu, Renming Hu, Yiming Li

**Affiliations:** ^1^Department of Endocrinology and Metabolism, Huashan Hospital, Fudan University, Shanghai 200040, China; ^2^Department of Endocrinology & Metabolism, Shanghai Institute of Endocrine and Metabolic Diseases, Ruijin Hospital, Shanghai Jiaotong University School of Medicine, Shanghai 200025, China

## Abstract

Idiopathic hypogonadotropic hypogonadism (IHH) is a rare condition in which puberty does not take place naturally. We aimed to develop and follow an internet-based cohort and to improve our understanding of the disease. We established an internet-based questionnaire survey. A total of 74 male IHH patients were recruited from the Chinese largest IHH network social group. The clinical symptoms before treatment mainly included small testis, underdeveloped secondary sexual characteristics, and sexual dysfunction. After treatment, the penis length, testicular volume, external genital organ development, pubic hair, beard, laryngeal prominence, erection, and spermatorrhea were improved significantly (*P* < 0.001). 18.9% of the patients completed fertility; however, more than half of the patients still complained of poor happiness and low physical strength. In addition, improvements in penis and pubic hair development, testosterone normalization and the physical strength in IHH patients who received gonadotropin and androgen replacement therapy were better than in those who received single gonadotropin therapy (*P* < 0.05 for all). In conclusion, disease-specific network investigation can be used as an alternative method of medical research for rare diseases. The results of our cross-sectional study showed the effectiveness of hormone replacement therapy for IHH and implied that gonadotropin and androgen replacement therapy may be superior to gonadotropin treatment alone.

## 1. Introduction 

Idiopathic hypogonadotropic hypogonadism (IHH) refers to gonadal dysgenesis due to deficiency of gonadotropin-releasing hormone (GnRH) release from the hypothalamus. The prevalence of IHH is about 1/10,000 in men and 1/50,000 in women with a M/F ratio of 4-5 : 1 [1]. The clinical picture is characterized by the absence of pubertal development and infertility [2]. Most patients sought medical consultation because of cryptorchidism or small penis. Since IHH is rare, the number of IHH patients is small in any one geographical area. It is often difficult for a single clinical center to perform comprehensive assessment of the disease due to the limited number of cases [3]. Internationally, large-scale clinical studies on this rare disease are usually conducted in the form of multicenter trials. However, these kinds of studies are time consuming and require large amount of resources as well as close coordination among study groups.

With the advances in information technology in the recent years, people have utilized networks for different research purposes. By December 2011, the number of internet users in China had exceeded five hundred million [4]. With more wide-spread availability of networks and the dramatic increase in the number of internet users, many patients are using internet as new means of understanding their diseases. They form disease-specific social groups to share their knowledge and experience spontaneously, which makes it possible for medical researchers to conduct medical research through networks [3, 5]. Network investigation is a new modality of means of medical research that can cover large populations with low investigation costs [6], especially for investigation of a rare disease in a particular network social group. It can collect large numbers of clinical cases within a short period for the research purpose. In the present study, we collected the clinical data of IHH patients in China by interviewing IHH patients through network questionnaire and their medical records. We compared the obtained data with those reported in previous studies in the literature for the purpose of improving our understanding, diagnosis, and treatment of IHH.

## 2. Methods

### 2.1. Patients and Methods

The Kallmann Liangjian Social Group is the largest IHH social networking group in China (QQ as the real-time network communication tool, ID7685615) with 103 members. We conducted survey research through a professional IHH investigation website (http://www.zsgl.net/r/). The items of the questionnaire investigation were designed by endocrinologists on the basis of the data obtained from literatures of IHH in China and abroad. The questionnaires were anonymous, and participation in the survey research was completely voluntary. The questionnaire consists of 64 questions and takes 8–11 minutes to complete. The information obtained through the questionnaire survey includes the following: demographic characteristics, family history, age, clinical symptoms and laboratory tests at onset of disease, particular therapeutic protocols (testosterone (T), human chorionic gonadotropin (HCG), human menopausal gonadotropin (HMG), luteinizing-hormone-releasing hormone (LHRH), or random combination of them), developmental stage of the testis and secondary sex characteristics, sexual function, level of sexual hormones, and quality-of-life (QoL) rating. Using the Tanner classification pictures [7], patients made a self-assessment of the development of the external genital organs, pubic hair, and breasts. QoL was self-assessed using a 0–10 scoring system. In the questionnaires, self-reported data was referred to the same standard, while the laboratory examination results and disease diagnosis were actually obtained from medical records. Online questionnaire survey submission included electronic consent as part of the website log-on process. This study was approved by the Institutional Review Board of Huashan Hospital in Shanghai, China. A total of 75 questionnaires answering all of the 64 questions were obtained online between October 2011 and March 2012. All patients were males. Except for a replicate case, 74 questionnaire forms were effective, with the effective rate being 98.7%. The remaining 29 patients did not participate in the survey.

### 2.2. Quality Control

All of the participants were asked to provide copies of their medical records; then, the records were examined by one endocrinologist, and only IHH patients were allowed to join the QQ group. All activities and exchanges involved in the investigation were voluntary and free of charge. Participants can submit the questionnaire survey only after all of the 64 questions were answered; thus, there was no missing data for the investigation. The computer recorded the IP address of each patient to avoid repetition. All participants had to hand in their medical records for IHH and provide their contact means such as the E-mail address and telephone number, so that the investigators could check whether or not the information that the participants provided was authentic. Finally, the computer system generated tables automatically, and the investigated data were stored in the data bank directly.

### 2.3. Statistical Analysis

All data were expressed as mean ± SD and percentage. Differences between continuous variable groups were compared by a nonparametric test. Differences between categorical variable groups were compared by Chi-square test or bilateral probability of Fisher's exact test. Scores for overall QoL and correlations of clinical parameters were analyzed by Spearman's correlation analysis. Data analysis was performed using SPSS16.0.

## 3. Results

### 3.1. Clinical and Biochemical Characteristics

All of the 74 patients participating in the IHH investigation were males with the mean age being 27.3 ± 3.4 years (ranging from 20 to 35 years). The mean age at disease onset was 15.2 ± 6.6 years, and the duration of the disease ranged from 1 to 25 years with a mean of 12.1 ± 7.2 years. The height of the 74 patients ranged from 157 to 188 cm with a mean of 174.4 ± 6.9 cm. The body mass index (BMI) was 17.99–32.11 kg/m^2^ (mean: 24.27 ± 3.73 kg/m^2^), belonging to overweight (BMI of 24 kg/m^2^ is used as cutoff point for the Chinese adults to define overweight) [8]. Of the 74 patients, 28 (37.8%) patients had a history of smoking (2 cigarettes per day on average). All patients had junior middle school or higher education level, 25 (33.8%) patients had senior middle school education, and 37 (50%) patients had college education. All of patients had normal intelligence and denied having a family history of IHH. All the 74 patients were diagnosed with IHH, and 84% of them were diagnosed in general hospitals, 13.5% in district hospitals, and 2.7% in community hospitals. 42 (56.8%) patients were diagnosed and treated in endocrinology unit, 25 (33.5%) patients in urinary surgery unit, and 6 (8.1%) patients in pediatrics unit. One patient was diagnosed in the reproductive clinic.

Of the 74 patients, 38 (51.4%) patients had hyposmia or anosmia, and 69 (93.2%) patients presented with obvious pubertal delay. The serum testosterone levels at disease onset were decreased with varying degrees in all of the 74 patients, as the medical reference ranges in different laboratories are different. Serum follicle-stimulating hormone (FSH) levels were decreased in 71 (95.9%) patients, and serum luteinizing hormone (LH) levels were decreased in 69 (93.2%) patients. Of the 74 patients, 68 patients received hormone replacement therapy, and the therapeutic protocols are shown in Table 1. 20 (27.0%) patients had cryptorchidism, and 11 patients received “cryptorchidism surgery.” Except for the family history, there was no significant difference in gender composition, age, BMI, testosterone level at disease onset, pubertal development, small penis, testicular volume, breast enlargement, cryptorchidism, and number of patients receiving “cryptorchidism surgery” between the populations in our study and the study reported by the Reproductive Endocrine Unit of Massachusetts General Hospital [9] (Table 2), while, in one multicenter study from England, more patients had family history of IHH, and the testicular volumes of IHH patients are smaller than those in our study [10].

### 3.2. Treatment Responses

In the 74 patients, the mean length of the penis before erection was 3.98 ± 1.73 cm, and the mean testicular volume was 4.11 ± 2.12 mL. None of the patients had normal external genital organs or pubic hair development. Based on the Tanner classification criteria, there are 38 patients at genital development stage I and 36 patients at stage II, and nobody was at stage III, IV, or V. There are 46 patients at pubic hair development stage I and 28 patients at stage II, and nobody was at stage III, IV, or V. All of the 74 patients had no or small laryngeal prominence and no or sparse facial hair including 12 (16.2%) patients with small laryngeal prominence and 9 (12.2%) with sparse beard. Of the 74 patients, only 27 (36.5%) patients had the male erection function, and 8 (10.8%) patients had physiological nocturnal spermatorrhea.

Among the 68 patients who received hormone replacement therapy, both their secondary sexual characteristics and the sexual function were improved markedly after treatment. For example, they had more masculine body, more facial hair and pubic hair, larger laryngeal prominence, more developed external genital organs, larger penis and scrotum, stronger erections, and nocturnal spermatorrhea. There was significant difference in penis length, testicular volume, development of external genital organs, pubic hair, beard, laryngeal prominence, penis erection, and spermatorrhea before and after treatment (*P* < 0.001, Table 3). The normalization of testosterone level rose from 0% before treatment to 66.2% after treatment, excluding 3 patients who are now receiving exogenous testosterone therapy. Of the 74 patients, 38 (51.4%) patients now have sexual activities, including 14 (18.9%) patients whose spouses became pregnant.

Thirty-seven patients who received HCG or HCG + HMG treatment were included into a single gonadotropin group (G group), and 25 patients who received HCG + T or HCG + HMG + T treatment were included into a gonadotropin + androgen replacement group (G + A group). Those who were still receiving testosterone replacement therapy were excluded from the G + A group, and the remaining 22 patients who once received testosterone replacement therapy discontinued its use for 1–8 years (mean: 4.5 ± 2.6 years). There was no significant difference in age at the time of investigation, age at disease onset, testosterone level before treatment, and decrease rate of FSH and LH between G group and G + A group. It was found that G + A group was significantly superior to G group in terms of penis length, pubic hair, laryngeal prominence development, and normalization of testosterone level (*P* < 0.05, Table 3). Compared with G group, G + A group also exhibited more improvement in testicular volume, genital organs, beard, and spermatorrhea, though the difference was not statistically significant.

### 3.3. Present QoL Scores

QoL of the IHH patients was assessed with respect to physical strength, appetite, sleep, libido, and sense of happiness. The mean score of physical strength was 5.1 ± 1.7, that of appetite was 7.3 ± 1.8, that of sleep was 6.8 ± 2.3, that of libido was 7.2 ± 2.0, and that of sense of happiness was 5.7 ± 2.6. The overall QoL scored was 10–45, and the mean value was 32.1 ± 7.5. The IHH patients reported that they still felt poor in the sense of happiness and physical strength after treatment. There was a significant difference in the physical strength score between G group and G + A group (5.40 ± 0.36 versus 4.65 ± 0.30, *P* = 0.025), while there was no significant difference in appetite, sleep, libido, and sense of happiness. The overall QoL score of the IHH patients was positively correlated with the level of testicular volume (*r* = 0.324;   *P* = 0.005) and the developmental state of the external genital organs (*r* = 0.281; *P* = 0.015) after treatment.

## 4. Discussion

IHH is a rare congenital sexual dysfunction disorder. The most unique characteristic of the present study is the utilization of a web-based disease-specific social networking group for our investigation. To the best of our knowledge, this is the first report conducting network-based analysis of IHH. The continuous expansion of internet users provides a good foundation for the development of network investigation, a new approach for medical research in China and elsewhere. There had been multiple studies comparing network studies with conventional studies, and they found that there was no significant difference in the study population characteristics and statistical results [11, 12]. Table 2 shows that the results obtained in the present network investigation are similar to those reported in the literature [9] with respect to sex, age, BMI, and clinical characteristics of the IHH patients, which further confirms that group data obtained from network investigation are representative. However, IHH patients in the study reported no family history, which may be related to the age and sporadic characteristics of the patients. The patients' parents did not have IHH, or else they would not have offspring as there was no domestic treatment method for IHH several decades ago. This finding is consistent with those of the previous large-sample studies on IHH in China [13]. Compared with conventional studies, network investigation has the following advantages. (1) It can include more participants, especially when network investigation is directed at a particular disease-specific social group. In addition, it is not restricted by time, space, or location (i.e., a single medical center), so that more patients with different severities and courses of the disease distributed in different regions of China or around the world can be investigated, and, therefore, the data obtained are more representative [3]. (2) Information can be obtained more quickly and conveniently, because network investigation saves amount of time in patient recruitment [14]. In this study, we only spent five months to collect the data of the 74 IHH patients compared with 20 years for the Massachusetts General Hospital [9]. In addition, network questionnaire investigation has a quick response rate, and the data obtained can be imported into the data bank directly, thus saving time, material, and manpower and reducing the cost of research effectively as compared with the conventional research methods [15]. (3) There is little difference in data collection. Network investigation can be completed in a relatively independent situation, devoid of external interferences, so that the patients are able to express freely their sexual function and other private information [3, 15]. (4) The requirement for network-based research is relatively low. Network investigation can be carried out by a relatively small and independent research team, and any clinician can conduct the research as long as he/she has a reasonable study design. Network investigation for rare diseases has unique advantages.

Although network investigation is a new method for medical research and carries many advantages, it has some disadvantages. (1) As only network users can participate in the study, there may be some bias in patients selection [16]. The participants need to be able to get on networks and have a certain education level. Therefore, patients with mental and cognitive impairment were automatically excluded. (2) Network investigation has to use the layman's language for communication, and, therefore, it is not suitable for studies that strongly depend on medical terminology [17].

The condition of IHH patients can be improved or reversed after hormone replacement therapy. Prolonged sex hormone deficiency may otherwise produce adverse effects on the development, metabolism, and psychology. Replacement therapy includes sex hormone replacement, gonadotropin, and GnRH pulse infusion [18]. The present study discovered six different therapeutic protocols (Table 1). All treated patients reported their improvements in the penis, testis, external genital organs, pubic hair, beard, laryngeal prominence development, erection, spermatorrhea, and testosterone normalization. These findings are basically consistent with those of the previous studies of US Massachusetts General Hospital and UK Manchester Royal Infirmary [8, 19]. More than 50% of the patients in this study have regained their sexual function after treatment, and part of the patients' spouses have got pregnant. However, the mammary glands of the IHH patients became even larger after treatment. The possible reason is that most of the IHH patients in our study were overweight; with physical development of the body and accumulation of the adipose tissue, the breasts became larger, which made self-assessment of the mammary development according to the Tanner classification criteria difficult. For this reason, ultrasonography should be used to evaluate mammary gland development more accurately. In addition, as the physical strength of IHH patients is generally weak and the sense of happiness is usually low, it is necessary to provide psychosocial support to IHH patients [20]. Unfortunately, there is a lack of such studies in both China and other countries.

In the present study, we interestingly found that the therapeutic effect in G + A group was superior to that in G group with respect to the penis length, pubic hair growth, testosterone normalization rate, and self-assessment scores of physical strength. The testosterone normalization rate in the G + A group (excluding the three patients who were still receiving testosterone replacement therapy in the recent three months) was significantly higher than that in G group (90.9% versus 51.7%), indicating that improvement of the clinical symptoms in G + A group was not due to the immediate exogenous testosterone treatment but due to the therapeutic effect of the testosterone treatment that they previously received. Whether exogenous testosterone replacement therapy is beneficial to the response of IHH patients to gonadotropin therapy remains inconclusive, but the report from Harvard Reproductive Endocrine Sciences Center seems to support this [21]. The conditions of IHH can be reversed in about 10% of the patients, and all of these reversed cases received androgen replacement therapy [21], although the course of treatment may vary from individual to individual. Therefore, they postulated that androgen may be able to improve the plasticity of the brain nerve network in generating GnRH, thus promoting reversal of the IHH condition. Androgen has been found in regulating the generation of neurons in human olfactory epithelium, olfactory bulb, and hippocampal dentate gyrus and maintaining a normal number of neuronal synapses [22]. In our investigation, 62/68 patients received exogenous HCG and/or HMG therapy, and the feedback inhibition of exogenous testosterone does not exist, while only 6 patients receiving LHRH may be mildly influenced. Another simple reason is that the HCG dose may not have been adequately titrated up, so androgen was being used as a kind of “top-up” therapy to increase spermatogenesis efficiency and fertility in the males. Similar to a recent study, recombinant FSH pretreatment followed by GnRH is successful in inducing testicular growth and fertility in men with congenital hypogonadotropic hypogonadism with prepubertal testes [23]. The mechanism(s) for the more favorable clinical outcome of G + A treatment over the simple G treatment cannot be elucidated in the present study. However, the phenomenon of “IHH reversal” as observed by Harvard Reproductive Endocrine Sciences Center suggests that testosterone treatment may improve the therapeutic effect of gonadotropin through an unknown pathway or mechanism in a particular stage [21]. Future randomized controlled clinical trials and mechanistic studies are needed before any definitive conclusions can be made.

In conclusion, disease-specific network investigation can be used as an alternative method of medical research, although its effectiveness needs to be further verified. In the present study, we made use of the network information technology to conduct an investigation on IHH and found that hormone replacement therapy could improve the conditions of IHH patients effectively. In addition, the commonly used gonadotropin + androgen therapy appears to be superior to androgen replacement alone, although the actual clinical significance needs to be confirmed in prospective randomized control studies.

## Figures and Tables

**Table 1:** Different therapeutic protocols that IHH patients received.

Therapeutic protocols	Number
Androgen (2 years) + HCG (3 years)	6
Androgen (8 years) + HCG (8 years)	3
Androgen (1 year) + HCG (2 years) + HMG (1 year)	1
Androgen (1 year) + HCG (1 year) + HMG (2 years)	3
Androgen (2 years) + HCG (1 year) + HMG (2 years)	3
Androgen (2 years) + HCG (3 years) + HMG (2 years)	3
Androgen (2 years) + HCG (7 years) + HMG (4 years)	2
Androgen (2 years) + HCG (7 years) + HMG (7 years)	4
Androgen (2 years) + HCG (17 years) + LHRH (3 years)	3
Androgen (1 year) + HCG (4 years) + HMG (2 years) + LHRH (2 years)	3
HCG (2 years)	3
HCG (3 years)	3
HCG (8 years)	1
HCG (2 years) + HMG (1 year)	11
HCG (2 years) + HMG (2 years)	7
HCG (3 years) + HMG (2 years)	8
HCG (4 years) + HMG (4 years)	3
HCG (10 years) + HMG (10 years)	1

Total	68

HCG: human chorionic gonadotropin; HMG: human menopausal gonadotropin; LHRH: luteinizing-hormone-releasing hormone.

**Table 2:** Comparison of general characteristics of IHH patients between the present network investigation and studies reported in the literature.

Study group	Present study single center	Nelly Pitteloud* single center	Richard Quinton** multicenter
Study period	5 months	20 years	25 years
Men (%)	74 (100)	78 (100)	170 (79.1)
Age at evaluation (yr)	27.3 ± 3.4	27 ± 0.7	NA
Family history (%)	0 (0)	19 (24.4)	52 (24.2)
Body mass index (kg/m^2^)	24.27 ± 3.73	25 ± 0.5	NA
Decreased testosterone (%)	74 (100)	78 (100)	170 (100)
Pubertal development (%)	5 (6.8)	6 (7.7)	NA
Small penis (%)	16 (21.6)	12 (15.4)	NA
Testicular volume (mL)	4.11 ± 2.12	6.1 ± 0.7	2.1 (1.9–2.3)
Breast enlargement (%)	26 (35.1)	26 (33.3)	NA
Cryptorchidism (%)	20 (27.0)	23 (29.5)	80 (49.7)
Cryptorchidism surgery (%)	17 (85.0)	67 (85.9)	55 (68.8)

*Reference [9].

**Reference [10].

**Table 3:** Characteristics of the 74 IHH patients, G group patients and G + A group patients.

	Total^a^	G group	G + A group^b^	P*
Number (*n*)	74	37	22	
Age (yr)	27.3 ± 3.4	27.16 ± 3.60	27.36 ± 2.7	0.821
Age at disease onset (yr)	15.2 ± 6.6	15.83 ± 7.06	14.46 ± 6.17	0.777
Decreased testosterone (%)	74 (100)	74 (100)	74 (100)	1.000
Decreased FSH (%)	71 (95.9)	37 (100)	22 (100)	1.000
Decreased LH (%)	69 (93.2)	37 (100)	22 (100)	1.000
Penis length (cm)				
Baseline	3.98 ± 1.73	3.65 ± 2.07	3.97 ± 1.31	0.135
After treatment	7.61 ± 2.63	6.91 ± 2.58	9.41 ± 2.26	0.002
Mean difference	3.64 ± 2.60	3.26 ± 2.74	5.43 ± 2.02	0.001
*P* value	<0.001	<0.001	<0.001	
Testicular volume (mL)				
Baseline	4.11 ± 2.12	4.32 ± 2.70	2.36 ± 1.87	0.215
After treatment	14.31 ± 6.6289	14.79 ± 8.49	13.61 ± 9.29	0.419
Mean difference	10.20 ± 5.82	10.47 ± 6.29	11.26 ± 5.37	0.243
*P* value	<0.001	<0.001	<0.001	
Genital development, Tanner stage (I/II/III/IV/V)				
Baseline	38/36/0/0/0	17/20/0/0/0	16/6/0/0	0.060
After treatment	1/14/38/19/2	1/8/17/10/1	0/3/17/2	0.613
*P* value	<0.001	<0.001	<0.001	
Pubic hair development, Tanner stage (I/II/III/IV/V)	
Baseline	46/28/0/0/0	18/19/0/0/0	16/6/0/0/0	0.103
After treatment	3/12/19/28/12	3/6/13/12/3	0/0/3/10/9	0.004
*P* value	<0.001	<0.001	<0.001	
Breast development, Tanner stage (I/II/III/IV/V)				
Baseline	18/30/19/7/0	6/15/12/4/0	6/10/3/3/0	0.404
After treatment	0/33/29/12/0	0/15/13/9/0	0/9/10/3/0	0.589
*P* value	<0.001	0.001	<0.001	
Beard (%)				
Baseline	9 (12.2)	5 (13.5)	3 (13.6)	0.989
After treatment	53 (71.6)	23 (62.2)	18 (81.8)	0.195
Mean difference	44 (59.5)	18 (48.6)	15 (68.2)	0.144
*P* value	<0.001	<0.001	<0.001	
Laryngeal prominence (%)				
Baseline	12 (16.2)	6 (16.2)	1 (4.5)	0.180
After treatment	49 (66.2)	21 (56.8)	16 (72.7)	0.220
Mean difference	37 (50.0)	15 (40.5)	15 (68.2)	0.040
*P* value	<0.001	0.001	<0.001	
Erection (%)				
Baseline	27 (36.5)	11 (29.7)	9 (40.9)	0.553
After treatment	67 (90.5)	33 (89.2)	19 (86.4)	0.745
Mean difference	40 (54.1)	22 (59.5)	10 (45.5)	0.439
*P* value	<0.001	<0.001	<0.001	
Spermatorrhea (%)				
Baseline	8 (10.8)	4 (10.8)	4 (18.2)	0.684
After treatment	44 (59.5)	17 (45.9)	17 (77.3)	0.037
Mean difference	36 (48.6)	13 (35.1)	13 (59.1)	0.073
*P* value	<0.001	0.002	<0.001	
Normalization of testosterone (%)				
Baseline	0 (0)	0 (0)	0 (0)	1.000
After treatment	47 (66.2)	19 (51.7)	20 (90.9)	0.004
*P* value	<0.001	<0.001	<0.001	

G group: patients receiving HCG or HCG + HMG treatment.

G + A group: patients receiving HCG + T or HCG + HMG + T treatment.

^a^For all 74 patients included in the study.

^b^For G + A group excluding 3 patients who were still receiving exogenous testosterone replacement.

*The difference between G group and G + A group.
